# Knowledge and thresholds for palliative care and surgery among healthcare providers caring for adults with serious illness

**DOI:** 10.3389/fmed.2024.1351864

**Published:** 2024-05-31

**Authors:** Darryl Wen Kai Juan, Irene Ai Ting Ng, Louis Choon Kit Wong, Wei Jing Fong, Piea Peng Lee, Sui An Lie, Jamie Xuelian Zhou, Mingzhe Cai, Johnny Chin-Ann Ong, Jane Chin Jin Seo, Claramae Shulyn Chia, Jolene Si Min Wong

**Affiliations:** ^1^Department of Sarcoma, Peritoneal and Rare Tumours (SPRinT), Division of Surgery and Surgical Oncology, National Cancer Centre Singapore, Singapore, Singapore; ^2^Department of Sarcoma, Peritoneal and Rare Tumours (SPRinT), Division of Surgery and Surgical Oncology, Singapore General Hospital, Singapore, Singapore; ^3^Division of Surgery and Surgical Oncology, National Cancer Centre Singapore, Singapore, Singapore; ^4^Division of Anaesthesiology, Singapore General Hospital, Singapore, Singapore; ^5^Division of Supportive and Palliative Care, National Cancer Centre, Singapore, Singapore; ^6^SingHealth Duke-NUS Surgery Academic Clinical Program, Duke-NUS Medical School, Singapore, Singapore; ^7^Laboratory of Applied Human Genetics, Division of Medical Sciences, National Cancer Centre Singapore, Singapore, Singapore; ^8^Institute of Molecular and Cell Biology, A^*^STAR Research Entities, Singapore, Singapore

**Keywords:** palliative care, serious illness, palliative surgery, end of life, palliative interventions

## Abstract

**Introduction:**

Timely palliative care and surgical interventions improve symptoms, health-related quality of life (HRQoL), and reduce medical cost for seriously ill adults at end of life (EOL). However, there is still poor delivery and underutilization of these palliative services. We hypothesize that the sub-optimal delivery is due to limited understanding among healthcare providers.

**Methods:**

A nationwide cross-sectional online survey was conducted among primary and tertiary healthcare providers. The survey assessed challenges faced, palliative education, confidence in managing palliative patients, and knowledge on palliative surgery. Overall palliative care awareness and knowledge was assessed using a 6-point score. Likelihood of considering various palliative interventions at EOL was also determined using a threshold score (higher score = higher threshold).

**Results:**

There were 145 healthcare providers who completed the survey (81.9% response rate); majority reported significant challenges in providing various aspects of palliative care: 57% (*n* = 82) in the provision of emotional support. Sixty-nine percent (*n* = 97) in managing social issues, and 71% (*n* = 103) in managing family expectations. Most expressed inadequate palliative care training in both under-graduate and post-graduate training and lack confidence in managing EOL issues. Up to 57% had misconceptions regarding potential benefits, morbidity and mortality after palliative surgery. In general, most providers had high thresholds for Intensive Care Unit admissions and palliative surgery, and were more likely to recommend endoscopic or interventional radiology procedures at EOL.

**Conclusion:**

Healthcare providers in Singapore have poor knowledge and misconceptions about palliative care and surgery. Improving awareness and education among those caring for seriously ill adults is essential.

## 1 Introduction

Timely integration of palliative care into the management of adults with serious illness have been shown to significantly improve symptoms, health-related quality of life (HRQoL) and is associated with less intensive medical care, reduced rates of acute healthcare utilization and costs at end of life (1, 2). Despite these well-established benefits, there is still poor delivery, underutilisation and delayed referral to palliative care services among seriously ill adults (3–5). Healthcare providers also continue to report barriers to offering palliative care and < 50% of seriously ill adults receive palliative care consults during their last year of life (6).

The field of palliative surgery faces similar challenges. While timely surgical interventions in appropriately selected adults have been shown to improve HRQoL and can effectively palliate troublesome symptoms, most physicians continue to have reservations toward invasive treatment during end of life (5, 7, 8). In fact, one of the major factors preventing the timely delivery of palliative surgery in suitable patients was misconceptions toward the indications for surgery among healthcare providers (9).

Inadequate knowledge and misconceptions regarding the risks and benefits of palliative surgery or other interventions at the end of life may contribute to the suboptimal delivery of palliative care, including resistance toward these interventions. As such we aim to evaluate common challenges faced in delivering palliative care, knowledge on palliative surgery and explore threshold for consideration of various palliative surgical interventions among local healthcare providers caring for seriously ill adults.

## 2 Materials and methods

### 2.1 Study design and participants

A nationwide cross-sectional online survey was conducted among primary and tertiary healthcare providers involved in the care of seriously ill adults between January and March 2022. This study was approved by SingHealth Centralized Institutional Review Board.

### 2.2 Study instrument and scoring

We developed a 34-item survey (Supplementary Table 1) following a review of literature and in consult with a multi-disciplinary palliative intervention team (10). We piloted the survey among members of the team to access comprehensibility and completion time (11).

The survey consisted of three sections:

(1) Palliative care knowledge, challenges, and confidence in managing end of life issues (11 items),
(2) Knowledge of palliative surgery and other interventions (six items), and
(3) Thresholds for considering various palliative interventions including surgery, endoscopy, interventional radiology (IR) procedure, Intensive Care Unit (ICU) admission during end of life (12 items).

We recorded survey participants' age, gender, type and years of practice, and proportion of seriously ill adults in their current clinical practice. The first section evaluated common palliative care related challenges encountered, perceived sufficiency of education in palliative knowledge, and confidence in the delivery of palliative care. The knowledge section assessed participants' knowledge regarding the definition, prevalence, rates of morbidity and mortality, as well as the function and intent of palliative surgery. Each item carried a score of one point for a total of six points, and a higher score indicative of higher knowledge levels on various aspects of palliative surgical interventions. The threshold section used a Likert scale to assess the likelihood of recommending different palliative interventions at different stages of end-of-life; response options ranged from 1 (Always Consider) to 5 (Never Consider). The total threshold score for each intervention type ranged from 5 to 20, with a low score indicative of a lower threshold or higher likelihood of considering that palliative intervention.

### 2.3 Statistical analysis

Descriptive statistics were used to summarize the demographic characteristics of the survey participants. Frequencies and percentages were calculated for categorical variables, while means, standard deviations (SD) and 95% confidence intervals were computed for continuous variables. All statistical analyses were carried out using the RStudio version 2022.02.3+492 with version 4.0.5 of R, and *P* < 0.05 was considered statistically significant.

## 3 Results

A total of 177 healthcare providers from 11 different subspecialties participated in the survey. Completed response rate was 81.9 % (*n* = 145). The mean age of the respondents was 39 years and 63.5% (*n* = 101) had more than 5 years of clinical practice. 75.6% (*n* = 118) reported clinical practices with a low (< 30%) volume of seriously ill adults. All other demographics of survey participants are shown in Table 1.

**Table 1:** Demographic characteristics of survey participants from 11 subspecialty departments involved in the care of seriously ill adults (*n* = 145).

	**No**.	**%**
**Age**		
20–30	26	15.1
31–40	93	54.1
41–50	30	17.4
>50	23	13.4
**Gender**		
Female	91	51.4
**Type of practice**		
Internal medicine specialist	33	18.6
Primary care physician	36	20.3
Surgeon	63	35.6
Palliative care physician	15	8.5
Nurses/allied health	30	16.9
**Years of practice**		
< 3 years	32	20.1
3–5 years	26	16.4
5–10 years	59	37.1
>10 years	42	26.4
**Proportion of adults seen in current clinical practice**		
**who are seriously ill (prognosis**<**1 year)**		
< 30%	118	75.6
30%−50%	12	7.7
50%−70%	4	2.6
>70%	22	14.1

### 3.1 Challenges, education, and confidence in providing palliative care

Survey participants reported the following common challenges in the care of seriously ill adults: providing emotional support (*n* = 82), addressing social issues (*n* = 97) and managing the expectations and needs of family members (*n* = 103; Table 2). Majority of participants expressed the lack of palliative care training at both under-graduate (*n* = 97) and post-graduate (*n* = 77) levels. Most also expressed a lack confidence in managing end of life issues, and conducting advanced care planning (ACP) discussions (Supplementary Table 2).

**Table 2:** Common challenges in the care of seriously ill adults during end of life.

	**No**.	**%**	***P*-value**
Controlling physical symptoms	81	55.9	0.1873
Communication of prognosis	73	50.3	
Advanced care planning	81	55.9	
Social issues	97	66.9	
Providing emotional support	82	56.6	
Deciding when to withhold and withdraw life-sustaining treatment e.g., chemotherapy/surgery/radiotherapy/ICU care etc.	85	58.6	
Managing the expectations and needs of patients' family members	103	71.0	

### 3.2 Knowledge of palliative surgery and other interventions

Mean aggregate knowledge score for palliative surgery was 3.38 out of six (range 1–6, 95% CI 3.19–3.54). 97.9% of participants were able correctly define palliative surgery as “Surgery performed to relief symptoms and improve quality of life amongst terminally ill patients” as defined by American College of Surgeon, and 90.3% correctly identified its purposes and intent (9). However, majority of participants were unable to accurately identify other important aspects of palliative surgery; 80.7% had misinformation on the prevalence of palliative surgeries performed, 57.2 and 55.9% on rates of major morbidity and in-hospital mortality following palliative surgery respectively, and 55.9% on the range of symptoms that can be alleviated through palliative surgery. Questions regarding palliative surgery and proportion of participants with correct responses are shown in Table 3.

**Table 3:** Number and proportion of participants with correct responses to knowledge questions regarding palliative surgery (*n* = 145).

**Items**	**Correct response**		***P*-value**
	**No**.	**%**	
1. Definition of palliative surgery	142	97.9	< 0.001
2. Prevalence of palliative surgeries at major tertiary hospitals	28	19.3	
3. Rate of major morbidity after palliative surgery^a^	62	42.8	
4. Rate of in-hospital mortality after palliative surgery	64	44.1	
5. Range of symptoms that palliative surgery can alleviate	64	44.1	
6. Purpose and intent of palliative surgery	131	90.3	

^a^Major morbidity based on Calvien-Dindo classification, grade 3 & above—major complications requiring radiological/endoscopic or surgical re-interventions or organ failure.

### 3.3 Threshold for palliative surgery and other interventions during end-of-life

The average threshold scores were calculated for various palliative interventions. The mean threshold scores for endoscopic or interventional radiology (IR) procedures, surgery, and intensive care unit (ICU) admission were 8.79, 10.44, and 12.29, respectively, indicating that participants were most likely to consider endoscopic or interventional radiology procedures, followed by surgery, and then ICU admission for seriously ill adults during end of life. This pattern did not vary based on patient prognosis (Supplementary Table 3).

## 4 Discussion

By 2030, approximately one in four Singaporeans will be above the age of 65 (12). Serious illness is common in an aging society, affecting 36% of older adults (13, 14). As such, palliative care which aims to improve quality of life in adults with life-limiting conditions, will play an increasingly important role. Unfortunately, in healthcare institutions worldwide, the lack of emphasis on education and training of healthcare providers in palliative care principles have led to significant barriers in the effective management of end of life issues in this vulnerable group (15). In our study, up to 53% of surveyed healthcare providers expressed a lack of palliative care education at both under- and post-graduate levels. Not surprisingly, most also lacked confidence in the provision of basic palliative care such as pain and symptom relief, and conducting advanced care planning (ACP). In the United States, the Association of American Medical Colleges (AAMC) recognizing the importance of palliative care education in non-palliative specialist practitioners, recommends the integration of basic palliative care competencies among medical students (16). At the post-graduate level, most residency programs also offer dedicated palliative care courses and rotations for residents to gain exposure to the principles of palliative care. However, in Singapore, there is a general lack of awareness on the importance of palliative care and efforts in end of life care education and training in non-palliative specialists or general practitioners are lacking (17).

In our survey, participants reported several challenges in the care of seriously ill adults. These include difficulties in the management of pain and other symptoms, communication of prognosis, carrying out Advanced Care Planning (ACP) discussions, and the management of psycho-social issues. By elucidating these concerns, our study provides key information to guide the development of targeted palliative care education programs in these areas.

Palliative surgery is performed in up to 20% of seriously ill adults and has been shown to provide effective relief of symptoms and improve HRQoL (5). However, when surveyed, most local healthcare providers had high thresholds for considering palliative surgery or other interventions regardless of overall prognosis. Our findings are echoed by McCahill et al. (9), who found that one of the major barriers preventing the optimal adoption of palliative surgery in suitable candidates is an overall reluctance due prevailing misconceptions on the risk-benefit ratios of invasive procedures during end of life among American physicians. Addressing these misinformation and entrenched misconceptions through targeted education programs will be essential to improve the quality of care for seriously ill patients in whom surgery or other interventions may be indicated.

Our study has several limitations. Firstly, the small sample size and involvement of only specific subspecialty physicians limits its generalisability. In addition, as convenient sampling was performed, the findings were subjected to response bias. Finally, while we had obtained group consensus and performed pilot testing of our survey, the survey instrument adopted was not validated. The absence of a validated tool for assessing the variables of interest may impact the reliability and accuracy of the collected data.

In conclusion, our study sheds light on prevailing deficiencies in palliative care education and training and misconceptions on palliative surgical care among healthcare providers in Singapore. Given our aging population, this calls for an urgent need to ramp up efforts in raising awareness and improving the reach of palliative educational programs among healthcare providers involved in the care of seriously ill adults.

## Data availability statement

The raw data supporting the conclusions of this article will be made available by the authors, without undue reservation.

## Ethics statement

The studies involving humans were approved by Singhealth Centralized Institutional Review Board. The studies were conducted in accordance with the local legislation and institutional requirements. The participants provided their written informed consent to participate in this study.

## Author contributions

DJ: Writing – review & editing, Writing – original draft, Validation, Resources, Project administration, Methodology, Investigation, Formal analysis, Data curation, Conceptualization. IN: Writing – review & editing, Writing – original draft, Validation, Resources, Project administration, Methodology, Investigation, Formal analysis, Data curation, Conceptualization. LW: Writing – review & editing, Validation, Supervision, Investigation, Data curation, Conceptualization. WF: Writing – review & editing, Resources, Project administration, Methodology, Investigation, Formal analysis, Conceptualization. PL: Writing – review & editing, Validation, Supervision, Investigation, Data curation, Conceptualization. SL: Writing – review & editing, Validation, Supervision, Investigation, Data curation, Conceptualization. JZ: Writing – review & editing, Validation, Supervision, Investigation, Data curation, Conceptualization. MC: Writing – review & editing, Validation, Supervision, Investigation, Data curation, Conceptualization. JO: Data curation, Conceptualization, Writing – review & editing, Validation, Supervision, Investigation. JS: Writing – review & editing, Validation, Supervision, Investigation, Data curation, Conceptualization. CC: Writing – review & editing, Validation, Supervision, Investigation, Funding acquisition, Data curation, Conceptualization. JW: Writing – review & editing, Writing – original draft, Validation, Supervision, Resources, Project administration, Methodology, Investigation, Funding acquisition, Formal analysis, Data curation, Conceptualization.
