# Corrigendum: Knowledge and thresholds for palliative care and surgery among healthcare providers caring for adults with serious illness

**DOI:** 10.3389/fmed.2024.1467096

**Published:** 2024-08-20

**Authors:** Darryl Wen Kai Juan, Irene Ai Ting Ng, Louis Choon Kit Wong, Wei Jing Fong, Piea Peng Lee, Sui An Lie, Jamie Xuelian Zhou, Mingzhe Cai, Johnny Chin-Ann Ong, Jane Chin Jin Seo, Claramae Shulyn Chia, Jolene Si Min Wong

**Affiliations:** ^1^Department of Sarcoma, Peritoneal and Rare Tumours (SPRinT), Division of Surgery and Surgical Oncology, National Cancer Centre Singapore, Singapore, Singapore; ^2^Department of Sarcoma, Peritoneal and Rare Tumours (SPRinT), Division of Surgery and Surgical Oncology, Singapore General Hospital, Singapore, Singapore; ^3^Division of Surgery and Surgical Oncology, National Cancer Centre Singapore, Singapore, Singapore; ^4^Division of Anaesthesiology, Singapore General Hospital, Singapore, Singapore; ^5^Division of Supportive and Palliative Care, National Cancer Centre, Singapore, Singapore; ^6^SingHealth Duke-NUS Surgery Academic Clinical Program, Duke-NUS Medical School, Singapore, Singapore; ^7^Laboratory of Applied Human Genetics, Division of Medical Sciences, National Cancer Centre Singapore, Singapore, Singapore; ^8^Institute of Molecular and Cell Biology, A^*^STAR Research Entities, Singapore, Singapore

**Keywords:** palliative care, serious illness, palliative surgery, end of life, palliative interventions

In the published article, there was an error in affiliation 6. Instead of “SingHealth Duke-NUS Oncology Academic Clinical Program, Duke-NUS Medical School, Singapore, Singapore”, it should be “SingHealth Duke-NUS Surgery Academic Clinical Program, Duke-NUS Medical School, Singapore, Singapore”.

In the published article, there was a mistake in the Funding statement. The whole paragraph under Funding statement is incorrect. The correct Funding statement appears below.

