# Supplementary material for: Knowledge and thresholds for palliative care and surgery among healthcare providers caring for adults with serious illness
Source: Front Med (Lausanne). 2024 May 31;11:1351864. doi: 10.3389/fmed.2024.1351864 (PMC11179431; doi:10.3389/fmed.2024.1351864)
Supplement: Supplementary file 1 [file Table_1.DOCX]

**Supplementary Table 1: Cross-Sectional Survey Questions**

**Section 1: Basic Demographics**

| **Item** | **Question** | **Option** |
| --- | --- | --- |
| 1 | Please indicate your age (in years) |  |
| 2 | Please indicate your gender | Male |
|  |  | Female |
| 3 | Please indicate your type of practice | Internal Medicine Specialist |
|  |  | Primary Care Physician |
|  |  | Surgeon |
|  |  | Palliative Care Physician |
|  |  | Nurses/Allied Health |
| 4 | Please indicate your years of practice | <3 years |
|  |  | 3-5 years |
|  |  | 5-10 years |
|  |  | >10 years |
| 5 | What is the proportion of terminally ill (prognosis < 1 year) patients you see in your current clinical practice? | <10% |
|  |  | 10 to 30% |
|  |  | 10 to 30% |
|  |  | 50to70% |
|  |  | >70% |

**Section 2: Challenges faced, Education and Confidence in Palliative Care**

| 1 | Are you confident with managing end-of-life issues for terminally ill patients under you care? | Not confident at all |
| --- | --- | --- |
|  |  | Not so confident |
|  |  | Somewhat confident |
|  |  | Confident |
|  |  | Very Confident |
| 2 | How confident are you in conducting Advanced Care Planning (ACP) discussions with patients under your care? | Not confident at all |
|  |  | Not so confident |
|  |  | Somewhat confident |
|  |  | Confident |
|  |  | Very Confident |
| 3 | Palliative care education you received in **medical school** was sufficient to ensure adequate management of **end of life issues** in patients under your care? | Strongly disagree |
|  |  | Disagree |
|  |  | Neutral |
|  |  | Agree |
|  |  | Strongly Agree |
| 4 | Palliative care education you received during **residency/post-graduate training** was sufficient to ensure adequate management of **end-of-life issues** in patients under your care? | Strongly disagree |
|  |  | Disagree |
|  |  | Neutral |
|  |  | Agree |
|  |  | Strongly Agree |
| 5 | Nutrition issue is often bring raised during end-of-life discussion | Strongly disagree |
|  |  | Disagree |
|  |  | Neutral |
|  |  | Agree |
|  |  | Strongly Agree |
| 6 | Palliative care education you received during **residency/post-graduate training** was sufficient to address **nutritional/hydration** queries by patients under your care | Strongly disagree |
|  |  | Disagree |
|  |  | Neutral |
|  |  | Agree |
|  |  | Strongly Agree |
| 7 | What are your challenges when managing a palliative patient with life-limiting disease (tick all that apply)? | Controlling physical symptoms |
|  |  | Communication of prognosis |
|  |  | Advanced care planning |
|  |  | Social issues |
|  |  | Providing emotional support |
|  |  | Deciding when to withhold & withdraw life-sustaining treatment e.g., chemotherapy/surgery/radiotherapy/ICU care etc. |
|  |  | Managing the patients’ family |
|  |  | Others: (free text) |
| 8 | When would you consider referring a patient to the specialist palliative care team? | At diagnosis of a life-limiting disease |
|  |  | At symptom onset due to a life-limiting disease |
|  |  | During predicted last 1-year of a life-limiting disease |
|  |  | During terminal phases (last 3 months) of a life-limiting disease |
|  |  | During the last weeks or days prior to demise |
|  |  | Others: (free text) |
| 9 | End-of-life discussions are best provided by the specialist palliative care team | Strongly disagree |
|  |  | Disagree |
|  |  | Neutral |
|  |  | Agree |
|  |  | Strongly Agree |
| 10 | Control of troublesome symptoms during end-of-life is best managed by the specialist palliative care team | Strongly disagree |
|  |  | Disagree |
|  |  | Neutral |
|  |  | Agree |
|  |  | Strongly Agree |
| 11 | Emotional and Psycho-social support during end-of-life is best provided by the specialist palliative care team | Strongly disagree |
|  |  | Disagree |
|  |  | Neutral |
|  |  | Agree |
|  |  | Strongly Agree |

**Section 3: Knowledge of Palliative Surgery and Other Interventions**

| 1 | In your opinion, what is palliative surgery? | Surgery performed to prolong life amongst terminally ill patients |
| --- | --- | --- |
|  |  | Surgery performed to relief symptoms and improve quality of life amongst terminally ill patients |
|  |  | Surgery performed as a bridge to chemotherapy/radiotherapy |
|  |  | Surgery performed to provide cure amongst the terminally ill |
|  |  | Others: (free text) |
| 2 | What do you think is the proportion of palliative intent surgery performed amongst surgical cases at a major tertiary hospital? | <20% |
|  |  | 20to40% |
|  |  | 40to60% |
|  |  | 60to80% |
|  |  | >80% |
| 3 | What do you think is the rate of **major morbidity** (Calvien-Dindo grade 3 & above - major complications requiring radiological/endoscopic or surgical re-interventions or organ failure) after palliative surgery? | <20% |
|  |  | 20to40% |
|  |  | 40to60% |
|  |  | 60to80% |
|  |  | >80% |
| 4 | What do you think is the rate of in-hospital **mortality** after palliative surgery? | <20% |
|  |  | 20to40% |
|  |  | 40to60% |
|  |  | 60to80% |
|  |  | >80% |
| 5 | Which of the following symptoms is least likely to resolve with palliative medical/symptomatic treatment (e.g. analgesia, anti-emetic) alone? | Dyspnea from pleural effusion |
|  |  | Distension and vomiting from peritoneal carcinomatosis |
|  |  | Pain from bone metastases |
|  |  | Nausea and vomiting from intra-cranial metastases |
| 6 | Quality of life outcomes are more important than survival and morbidity outcomes when it comes to palliative intent surgery & interventions | Strongly disagree |
|  |  | Disagree |
|  |  | Neutral |
|  |  | Agree |
|  |  | Strongly Agree |

**Section 4: Threshold for Palliative Interventions**

| 1 | Please answer this question in accordance with the estimated life expectancy of a palliative patient. **Surgical procedures** should be avoided in patients with: |  |
| --- | --- | --- |
| a) | **Limited prognosis of <3 months** | Strongly disagree |
|  |  | Disagree |
|  |  | Neutral |
|  |  | Agree |
|  |  | Strongly Agree |
| b) | **Prognosis of 3 to 6 months** | Strongly disagree |
|  |  | Disagree |
|  |  | Neutral |
|  |  | Agree |
|  |  | Strongly Agree |
| c) | **Prognosis of 6 to 12 months** | Strongly disagree |
|  |  | Disagree |
|  |  | Neutral |
|  |  | Agree |
|  |  | Strongly Agree |
| d) | **Prognosis > 1 year** | Strongly disagree |
|  |  | Disagree |
|  |  | Neutral |
|  |  | Agree |
|  |  | Strongly Agree |
| 2 | Please answer this question in accordance with the estimated life expectancy of a palliative patient. **Endoscopic or Interventional radiological procedures** should be avoided in patients with |  |
| a) | **Limited prognosis of <3 months** | Strongly disagree |
|  |  | Disagree |
|  |  | Neutral |
|  |  | Agree |
|  |  | Strongly Agree |
| b) | **Prognosis of 3 to 6 months** | Strongly disagree |
|  |  | Disagree |
|  |  | Neutral |
|  |  | Agree |
|  |  | Strongly Agree |
| c) | **Prognosis of 6 to 12 months** | Strongly disagree |
|  |  | Disagree |
|  |  | Neutral |
|  |  | Agree |
|  |  | Strongly Agree |
| d) | **Prognosis > 1 year** | Strongly disagree |
|  |  | Disagree |
|  |  | Neutral |
|  |  | Agree |
|  |  | Strongly Agree |
| 3. | Please answer this question in accordance with the estimated life expectancy of a palliative patient. **ICU admission** for life-sustaining measures (defined as intubation, mechanical ventilation, vasopressors, dialysis) are acceptable for patients with |  |
| a) | **Limited prognosis of <3 months** | Strongly disagree |
|  |  | Disagree |
|  |  | Neutral |
|  |  | Agree |
|  |  | Strongly Agree |
| b) | **Prognosis of 3 to 6 months** | Strongly disagree |
|  |  | Disagree |
|  |  | Neutral |
|  |  | Agree |
|  |  | Strongly Agree |
| c) | **Prognosis of 6 to 12 months** | Strongly disagree |
|  |  | Disagree |
|  |  | Neutral |
|  |  | Agree |
|  |  | Strongly Agree |
| d) | **Prognosis > 1 year** | Strongly disagree |
|  |  | Disagree |
|  |  | Neutral |
|  |  | Agree |
|  |  | Strongly Agree |
