# Supplementary material for: Knowledge and thresholds for palliative care and surgery among healthcare providers caring for adults with serious illness
Source: Front Med (Lausanne). 2024 May 31;11:1351864. doi: 10.3389/fmed.2024.1351864 (PMC11179431; doi:10.3389/fmed.2024.1351864)
Supplement: Supplementary file 2 [file Table_2.DOCX]

Supplementary Table 2: Perceived sufficiency of palliative care education & Confidence level in providing palliative care (n=145)

|  | Strongly Disagree | | Disagree | | Neutral | | Agree | | Strongly Agree | | P |
| --- | --- | --- | --- | --- | --- | --- | --- | --- | --- | --- | --- |
|  | N | % | N | % | N | % | N | % | N | % |  |
| Palliative care education you received in medical school was sufficient to ensure adequate management of end-of-life issues in patients under your care? | 33 | 21.6 | 70 | 45.8 | 35 | 22.9 | 15 | 9.8 | 0 | 0.0 | <0.001 |
| Palliative care education you received during residency/post-graduate training was sufficient to ensure adequate management of end-of-life issues in patients under your care? | 17 | 11.0 | 65 | 41.9 | 30 | 19.4 | 37 | 23.9 | 6 | 3.9 | < 0.001 |
|  | **Not confident at all** | | **Not so confident** | | **Somewhat confident** | | **Confident** | | **Very Confident** | | **P** |
|  | N | % | N | % | N | % | N | % | N | % |  |
| Are you confident with managing end-of-life issues for terminally ill patients under your care? | 7 | 4.5 | 38 | 24.4 | 59 | 37.8 | 36 | 23.1 | 16 | 10.3 | <0.001 |
| Are you confident in conducting Advanced Care Planning (ACP) discussions with patients under your care? | 20 | 12.9 | 47 | 30.3 | 45 | 29.0 | 33 | 21.1 | 10 | 6.5 | <0.001 |
