# Supplementary material for: Knowledge and thresholds for palliative care and surgery among healthcare providers caring for adults with serious illness
Source: Front Med (Lausanne). 2024 May 31;11:1351864. doi: 10.3389/fmed.2024.1351864 (PMC11179431; doi:10.3389/fmed.2024.1351864)
Supplement: Supplementary file 3 [file Table_3.DOCX]

Supplementary Table 3: Mean threshold score for different types of palliative interventions at varying patient prognosis

| Prognosis | Intervention type | | |
| --- | --- | --- | --- |
|  | Surgical Procedures | Endoscopic/  Interventional radiological procedures | ICU Admission/ Life sustaining measures |
|  | Mean (SD) | | |
| <3 months | 3.51 (1.11) | 2.78 (1.12) | 3.37 (1.30) |
| 3-6months | 2.83 (0.953) | 2.28 (0.913) | 3.24 (1.04) |
| 6-12months | 2.28 (0.854) | 2.01 (0.901) | 2.97 (0.909) |
| >12 months | 1.82 (0.879) | 1.72 (0.878) | 2.71 (1.18) |
| Total | 2.61 (1.14) | 2.20 (1.03) | 3.07 (1.14) |
